# Metaplastic Breast Carcinoma in a 37-Year-Old Female: A Case Report

**DOI:** 10.7759/cureus.21881

**Published:** 2022-02-03

**Authors:** Inês Leão, David Afonso-João, Joana Esteves, Fernanda Fernandes, Ana Joaquim

**Affiliations:** 1 Medical Oncology, Centro Hospitalar Vila Nova de Gaia/Espinho, Vila Nova de Gaia, PRT; 2 Pathology, Centro Hospitalar Vila Nova de Gaia/Espinho, Vila Nova de Gaia, PRT; 3 General Surgery, Centro Hospitalar Vila Nova de Gaia/Espinho, Vila Nova de Gaia, PRT

**Keywords:** breast cancer, case report, central nervous system metastasis, young patient, metaplastic breast carcinoma

## Abstract

Metaplastic breast carcinoma (MBC) is a rare and aggressive histologic subtype of cancer. Because of its rarity and heterogeneity, the management of these patients is challenging. Here, we present the case of a rapidly progressive MBC with mesenchymal differentiation in a 37-year-old female, treated with trimodal therapy consisting of neoadjuvant chemotherapy with paclitaxel and carboplatin, followed by dose-dense cyclophosphamide and doxorubicin (ddAC), modified radical left mastectomy, and adjuvant radiotherapy. Despite the need to anticipate the surgery after the first cycle of ddAC, because of a life-treating adverse event, there was a pathologic complete response. Nevertheless, 6.2 months after completing adjuvant radiotherapy, the patient had a recurrence on the central nervous system (CNS) (two lesions), which was managed with excisional biopsy and stereotactic body radiation therapy. The patient also started “complementary” chemotherapy with capecitabine. Still, 18 months after being diagnosed, she died due to CNS disease progression.

## Introduction

Non-epithelial tumors of the breast are rare. Metaplastic breast carcinoma (MBC) comprises less than 1% of all invasive breast cancer tumors and is associated with poor prognosis [1,2]. Because of its rarity and heterogeneity, the management of these patients is challenging. Recent data suggest that trimodal therapy consisting of surgery, chemotherapy, and radiotherapy improves overall survival, although details regarding specific chemotherapeutic agents are lacking [3]. In the absence of randomized clinical trials, there is an urgent need for clinicians to share their experiences. Here, we present the case of a rapidly progressive MBC with mesenchymal differentiation in a 37-year-old female, with pathologic complete response after neoadjuvant chemotherapy but with a recurrence on the central nervous system 13 months after the diagnosis.

## Case presentation

**Figure 1:**
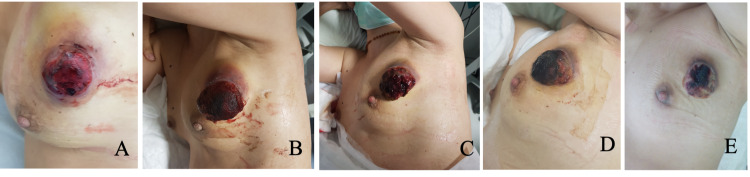
A: Tumor evaluation at the first medical oncology consultation. B–E: Tumor response assessment during neoadjuvant chemotherapy.

A fit and well 37-year-old female with no relevant medical and family history was referred to the medical oncology department after being diagnosed with a locally advanced MBC with mesenchymal differentiation. She had detected a rapidly growing lump in her left breast four months earlier, just two months after giving birth. On physical examination, there was a hard, painless mass located on the upper outer quadrant of the left breast, measuring approximately 100 mm in diameter and presenting an exophytic friable component measuring 40 × 40 × 20 mm (Figure 1A). The ipsilateral axilla was occupied by conglomerate lymph nodes.

**Figure 2:**
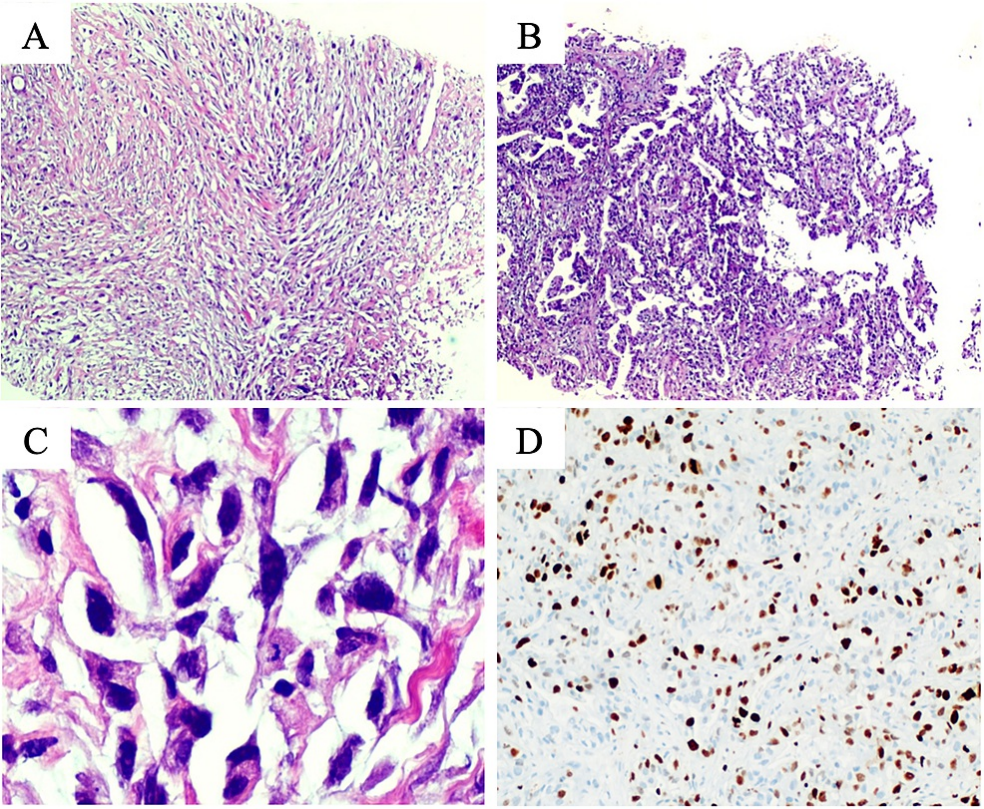
Pathologic findings of breast biopsy. On hematoxylin and eosin staining (H&E, 40×), a malignant epithelial neoplasm composed of fascicular areas of fusiform cells was found (A). Some other areas assumed more of a tubular or glandular architecture of epithelioid cells with abundant eosinophilic cytoplasm (B). On higher power (H&E, 400×), cytologic atypia was striking, with marked cellular pleomorphism, anisokaryosis, and frequent atypical mitotic figures (C). Immunostaining showed evidence of a highly proliferative malignancy, with a Ki-67 proliferative index above 30% (D).

At the time of referral to medical oncology, the patient had already undergone several investigation tests. Four months earlier, she was observed by her primary physician because of a small painful lump on her left breast. The first ultrasound examination revealed a hypoechoic solid lesion measuring 20 mm in diameter located on the upper outer quadrant of the left breast, suggestive of fibroadenoma. However, as the lump continued to grow, two months later, a second ultrasound examination was performed, which confirmed that the lesion had doubled in size (46 mm) and became more heterogeneous; moreover, it was now evident that it was adenomegaly measuring 17 mm in diameter in the ipsilateral axilla. Biopsy of both lesions led to the diagnosis of MBC with mesenchymal differentiation (carcinosarcoma). Immunohistochemical staining was performed on paraffin-embedded blocks of tumor tissue, and the carcinomatous epithelial cells displayed a positive reaction for cytokeratin (AE1, 14, and MNF), vimentin, and Ki-67 (superior to 30% of cells) and a negative reaction for ER, PR, and HER2neu; the mesenchymal cells exhibited a positive reaction for p63 (focal), vimentin, and CD10 (Figure 2).

Following a multidisciplinary team meeting, a metastatic workup was obtained. Nuclear medicine bone scan and CT of the thorax, abdomen, and pelvis showed no evidence of metastatic disease. Laboratory data showed raised Ca15.3 (47.5 U/mL) and normal CEA (0.5 ng/mL) with normal hemogram and renal function. The genetic test did not detect any known pathogenic variant on BRCA1, BRCA2, PALB2, ATM, and CHEK2 genes.

Being a triple-negative breast cancer, staged as cT4bN2M0 (AJCC TNM staging eighth edition (2017)), the patient was proposed for neoadjuvant chemotherapy with weekly paclitaxel 80 mg/m^2^ and carboplatin AUC 6 every three weeks (four cycles), followed by dose-dense cyclophosphamide 600 mg/m² and doxorubicin 60 mg/m² (ddAC) every two weeks (four cycles with eight days of prophylactic filgrastim). Prior to the beginning of treatment, an echocardiogram was obtained, and her ejection fraction was estimated to be 62% with normal left ventricle wall motion. Tumor response was closely monitored during the entire treatment. There was a rapid clinical response (shrinking and softening) since the first cycle (Figure 1B-1E).

After the first cycle of dose-dense cyclophosphamide and doxorubicin, the patient was diagnosed with pancytopenia (anemia grade 2, neutropenia, and thrombocytopenia grade 4) complicated with febrile neutropenia grade 4. At the time, she only referred a mild and generalized abdominal pain, and on physical examination, she was prostrated and hypotensive, and no focus of infection was identified. The patient was admitted to home hospitalization and treated with piperacillin/tazobactam for seven days and filgrastim for three days, with full recovery. In the multidisciplinary group meeting, it was decided to suspend chemotherapy and schedule surgery. Less than four weeks after the last chemotherapy administration (a total of five cycles), the patient was submitted to a modified radical left mastectomy. Pathology examination showed a complete pathologic response ypT0N0, with response signs in one of the 22 removed lymph nodes. Less than eight weeks after surgery, she underwent adjuvant radiotherapy to the chest wall, supraclavicular/infraclavicular regions, and internal mammary chain (50 Gy in 25 daily fractions delivered over five weeks).

Thirteen months after diagnosis, the patient was admitted to the emergency department due to an episode of a generalized tonic-clonic seizure. Physical examination revealed stable vital signs and no signs of head trauma or neurologic deficits. The cerebral CT revealed an intra-axial lesion measuring 25 × 18 mm located on the right frontal lobe, suggestive of metastasis. An MRI was later performed and confirmed the presence of the suspicious lesion measuring 22 × 25 × 20 mm on the right frontal lobe and revealed a second lesion measuring 9 × 10 × 7.5 mm on the left temporal lobe near the skull base (Figure 2).

As bone scan and CT of the thorax, abdomen, and pelvis showed no evidence of metastatic disease, the patient was submitted to excisional biopsy of the temporal lesion and stereotactic body radiation therapy (left temporal lesion: 30 Gy in five fractions for one week (VMAT); surgical site on right frontal lobe: 27 Gy in three fractions for 0.5 weeks (VMAT)). Pathology examination was consistent with breast cancer metastasis.

After completing the radiotherapy, the patient started “complementary” chemotherapy with capecitabine 1250 mg/m^2^ twice per day on days 1 to 14 every three weeks. However, after three cycles of treatment, with good clinical and hematological tolerability and no neurologic sequels, the patient was admitted to the emergency department with intense headaches. The cerebral CT scan revealed an increase of the lesion located on the frontal lobe (32 × 30 mm), with more vasogenic edema and associated hydrocephaly. The patient was admitted to an intermediate care unit and initiated high-dose corticotherapy. Nevertheless, the patient died that same night, 18 months after being diagnosed with metaplastic breast cancer.

## Discussion

MBC is a rare and heterogeneous histologic type of breast cancer that exhibits the transformation of part or all of its glandular carcinomatous component into a non-glandular or metaplastic component [1]. The World Health Organization currently recognizes five variants of metaplastic carcinoma based on their histologic appearance: low-grade adenosquamous carcinoma, fibromatosis-like metaplastic carcinoma, spindle cell carcinoma, squamous cell carcinoma, and with heterologous mesenchymal differentiation [4]. Most of these tumors are triple-negative (hormone receptor and HER2 negative) [1]. The median age at presentation for patients with MBC is 59 years [2]. Characteristically, patients with MBC present clinically with masses greater than 2 cm and with no axillary lymph node metastasis [2].

As MBC was not officially recognized as a distinct pathologic diagnosis until 2000, knowledge about treatment patterns and outcomes is limited. Recent studies reported that metaplastic breast carcinomas are associated with poor prognosis compared with other histologic subtypes [5-7]. The most recent international guidelines do not differentiate the treatment of patients with MBC from other breast-invasive tumors [8,9]. However, due to evidence of chemoresistance, the role of multimodal therapy remains an area of active investigation with growing evidence [3].

In recent years, neoadjuvant chemotherapy has been increasingly used as part of the multidisciplinary management of breast cancer. This treatment strategy allows the evaluation of tumor response to specific chemotherapy regimens, and complete pathologic response after neoadjuvant chemotherapy has been shown to be an independent predictor for survival compared with cases that fail to achieve such response [10,11]. There is a lack of studies evaluating the response of neoadjuvant chemotherapy in MBC. A recent retrospective study estimated a pathologic complete response of 17% (five of 29), being adriamycin, cyclophosphamide, and taxane (16 of 29) the most commonly used chemotherapy regiment [12]. The heterogeneity of patients included and the small sample size are important limitations.

Our clinical case demonstrates the excellent response to neoadjuvant chemotherapy (mainly to paclitaxel and carboplatin) of an aggressive locally advanced MBC with mesenchymal differentiation. The inclusion of platinum agents as neoadjuvant chemotherapy for triple-negative breast cancer is controversial, and we decided to incorporate this compound due to the urgent need for local tumor control [8]. Nevertheless, the patient had an early recurrence in the central nervous system, both lesions being treated radically. The decision to complement this therapeutic approach with eight cycles of capecitabine took into consideration the fact that, despite poor penetration by chemotherapy into the CNS under physiologic conditions, the blood-brain barrier is frequently dysfunctional within brain metastases [13]. Furthermore, it could prevent the development of metastasis outside the brain. Despite the strong recommendation to maintain systemic therapy and treat the central nervous system in case of disease progression only in the brain, there is a lack of evidence in how to proceed in case of a localized recurrence on the central nervous system.

Regarding subsequent treatment strategy, in case of progression, due to the limitations in the medical management of MBC, molecular profiling has been initiated to elucidate targeted therapies. Recently, small studies identified frequent mutations in PIK3CA (42%-48%) [14,15] and PD-L1 overexpression (50%) [15], both associated with worse prognosis [15] but potential target therapies. Unfortunately, the patient suddenly died probably due to a disease progression on the central nervous system, with no evidence of disease outside the brain.

## Conclusions

There is a lack of evidence on the management of locally advanced MBC. As suggested by international guidelines, the treatment plans for MBC according to the hormone receptor and HER2 classification are effective. This report highlights the aggressive subtype of MBC with a poor prognosis even in patients with a complete pathological response. Nevertheless, the fast and complete response to neoadjuvant treatment support this strategy.

## References

[1] McMullen ER, Zoumberos NA, Kleer CG (2019). Metaplastic breast carcinoma: update on histopathology and molecular alterations. Arch Pathol Lab Med.

[2] Li Y, Zhang N, Zhang H, Yang Q (2019). Comparative prognostic analysis for triple-negative breast cancer with metaplastic and invasive ductal carcinoma. J Clin Pathol.

[3] Kennedy WR, Gabani P, Acharya S, Thomas MA, Zoberi I (2019). Clinical outcomes and patterns of care in the treatment of carcinosarcoma of the breast. Cancer Med.

[4] World Health Organization (2019). Breast tumours. WHO classification of tumours, 5th edition.

[5] Sanges F, Floris M, Cossu-Rocca P (2020). Histologic subtyping affecting outcome of triple negative breast cancer: a large Sardinian population-based analysis. BMC Cancer.

[6] Bae SY, Lee SK, Koo MY (2011). The prognoses of metaplastic breast cancer patients compared to those of triple-negative breast cancer patients. Breast Cancer Res Treat.

[7] Ong CT, Campbell BM, Thomas SM (2018). Metaplastic breast cancer treatment and outcomes in 2500 patients: a retrospective analysis of a national oncology database. Ann Surg Oncol.

[8] (2020). National Comprehensive Cancer Network: NCCN clinical practice guidelines in oncology breast cancer. NCCN Clinical Practice Guidelines in Oncology Breast Cancer. National Comprehensive Cancer Network.

[9] Senkus E, Kyriakides S, Ohno S (2015). Primary breast cancer: ESMO Clinical Practice Guidelines for diagnosis, treatment and follow-up. Ann Oncol.

[10] Cortazar P, Zhang L, Untch M (2014). Pathological complete response and long-term clinical benefit in breast cancer: the CTNeoBC pooled analysis. Lancet.

[11] Bonnefoi H, Litière S, Piccart M (2014). Pathological complete response after neoadjuvant chemotherapy is an independent predictive factor irrespective of simplified breast cancer intrinsic subtypes: a landmark and two-step approach analyses from the EORTC 10994/BIG 1-00 phase III trial. Ann Oncol.

[12] Han M, Salamat A, Zhu L (2019). Metaplastic breast carcinoma: a clinical-pathologic study of 97 cases with subset analysis of response to neoadjuvant chemotherapy. Mod Pathol.

[13] Lin NU, Bellon JR, Winer EP (2004). CNS metastases in breast cancer. J Clin Oncol.

[14] Edenfield J, Schammel C, Collins J, Schammel D, Edenfield WJ (2017). Metaplastic breast cancer: molecular typing and identification of potential targeted therapies at a single institution. Clin Breast Cancer.

[15] Afkhami M, Schmolze D, Yost SE (2019). Mutation and immune profiling of metaplastic breast cancer: correlation with survival. PLoS One.

